# Protective Measures against COVID-19: Dental Practice and Infection Control

**DOI:** 10.3390/healthcare9060679

**Published:** 2021-06-04

**Authors:** Sri Nitya Reddy Induri, Yunah Caroline Chun, Joonmo Christopher Chun, Kenneth E. Fleisher, Robert S. Glickman, Fangxi Xu, Efthimia Ioannidou, Xin Li, Deepak Saxena

**Affiliations:** 1 Department of Molecular Pathobiology, College of Dentistry, New York University, New York, NY 10010, USA; si2067@nyu.edu (S.N.R.I.); ync226@nyu.edu (Y.C.C.); fx363@nyu.edu (F.X.); xl15@nyu.edu (X.L.); 2 College of Natural Sciences, University of Texas at Austin, Austin, TX 78712, USA; joonmochun@utexas.edu; 3 Department of Oral and Maxillofacial Surgery, College of Dentistry, New York University, New York, NY 10010, USA; kef3@nyu.edu (K.E.F.); rsg1@nyu.edu (R.S.G.); 4 Division of Periodontology, School of Dental Medicine, UCONN Health, Farmington, CT 06030, USA; ioannidou@uchc.edu; 5 Department of Surgery, School of Medicine, New York University, New York, NY 10016, USA

**Keywords:** COVID-19, SARS-2, dentistry, infection control, coronavirus, disinfection

## Abstract

The onset of the Coronavirus 2019 (COVID-19) pandemic has challenged the worldwide healthcare sector, including dentistry. The highly infectious nature of severe acute respiratory syndrome coronavirus 2 (SARS-CoV-2) virus and risk of transmission through aerosol generating procedures has profoundly impacted the delivery of dental care services globally. As dental practices with renewed infection control strategies and preventive measures are re-opening in the “new normal” period, it is the responsibility of healthcare professionals to constantly analyze new data and limit the spread of COVID-19 in dental care settings. In the light of new variants of SARS-CoV-2 rapidly emerging in different geographic locations, there is an urgent need to comply more than ever with the rigorous public health measures to mitigate COVID-19 transmission. The aim of this article is to provide dental clinicians with essential information regarding the spread of SARS-CoV-2 virus and protective measures against COVID-19 transmission in dental facilities. We complied and provided guidance and standard protocols recommended by credible national and international organizations. This review will serve as an aid to navigating through this unprecedented time with ease. Here we reviewed the available literature recommended for the best current practices that must be taken for a dental office to function safely and successfully.

## 1. Introduction

Currently, the rapidly escalating outbreak of 2019 novel Coronavirus (COVID-19) caused by severe acute respiratory syndrome coronavirus-2 (SARS-CoV-2) presents serious global concerns that are impacting public health, overall healthcare systems, and the global economy. The Hubei Province of China reported the first patient with pneumonia of unknown cause, resembling viral pneumonia on 31 December 2019 [1], and this pneumonia was designated as COVID-19 [2] and officially termed SARS-CoV-2 [3]. Due to the alarming spread and severity of SARS-CoV-2 worldwide, the World Health Organization (WHO) declared the outbreak as a Public Health Emergency of International Concern (PHEIC) on 30 January 2020 [4]. As of 21 April 2021, a total of 143 million cases have been confirmed globally, and 3 million deaths have been reported in at least 192 countries [5]. Strict measures are being extensively undertaken to mitigate the community transmission of COVID-19 through social distancing, quarantines, lockdowns, travel restrictions, and the requisite of wearing facemasks in public.

The transmission patterns of SARS-CoV-2 suggest that respiratory droplets and direct contact are the main routes of transmission [6]. With this notion, the possibility of aerosol transmission in a closed environment with high concentrations of vital aerosols is possible [7]. This type of transmission constitutes a special implication to dentistry as many dental procedures and tools used can induce or generate contaminated aerosols with the potential to cause infection [8]. Dental offices can act as focal spots of cross-contamination and can lead to nosocomial COVID-19 transmission to both patients and dental staff. In fact, a clinical study conducted in Wuhan, China found that of 138 patients, the human-to-human hospital related COVID-19 transmission was 41.3%, 29% of which occurred in healthcare workers [9]. Healthcare workers, including dentists and dental staff, who are on the frontline battling the COVID-19 pandemic, face an increase in the risk of contracting and transmitting the infection while delivering dental services.

The article aims to provide dental practitioners with essential knowledge concerning SARS-CoV-2 spread and protective measures against COVID-19 transmission. Here we reviewed available literature about COVID-19 and compiled the relevant guidance and standard-of-care recommended by public health organizations. 

## 2. SARS-CoV-2

The novel SARS-CoV-2 is an enveloped, positive sense single-stranded RNA virus belonging to the genera beta-coronaviruses [10] of the family *Coronaviridae* [11]. Data suggest that although bats were the initial reservoir of SARS-CoV-2, the transmission to humans was facilitated through intermediate hosts [12], which were most likely pangolins [13].

The clinical manifestations of COVID-19 are diverse with variable progression patterns that are still being evaluated and analyzed. The National Institute of Health (NIH) has categorized COVID-19 based on its illness severity: (1) asymptomatic or pre-symptomatic infection, (2) mild, (3) moderate, and (4) severe [14]. The dominant clinical symptoms are fever (>38.0 °C), dry cough, myalgia [15,16], fatigue, sore throat, and shortness of breath [16]. Less common symptoms include headache, sputum production, nausea, vomiting, nasal congestion, diarrhea, hemoptysis, and conjunctival congestion [15,17]. In children and adolescents, recent data report the occurrence of multisystem inflammatory syndrome in children (MIS-C) [18]/Pediatric Multisystem Inflammatory Syndrome (PIMS) temporally associated with COVID-19 [19]. Clinical presentation routinely includes several issues: (1) Kawasaki-like disease, (2) Kawasaki disease shock syndrome, (3) myocarditis, and/or (4) gastrointestinal symptoms [20]. The full spectrum of the clinical characteristics of COVID-19 is not conclusive as new information regarding the disease is continuously emerging.

The incubation period for SARS-CoV-2 ranges between 1 and 14 days (~2 weeks) with a mean incubation period of 5.1 days. Most individuals (97.5%) who show clinical symptoms develop the symptoms within 11.5 days [21]. Viral shedding is reported to begin 1–3 days before symptoms occur and the patient generally becomes most infectious while symptomatic [22]. Recent data report that no statistical difference in the COVID-19 viral load among those who are symptomatic versus those who are asymptomatic has been found [23]. One study has indicated that 50% of the overall COVID-19 outbreak is caused during pre-symptomatic and asymptomatic stages [24]. The data on asymptomatic SARS-CoV-2 transmission are currently limited; however, research addressing its transmission dynamics is progressing.

SARS-CoV-2 poses a greater risk to the elderly and especially to those with chronic coexisting illnesses who have compromised immune responses [25]. Risk factors for severe infection include obesity (body mass index [BMI] ≥ 28 kg/m^2^), type 2 diabetes, hypertension, elevated lactate dehydrogenase (LDH > 250 U/L), C-reactive protein (CRP > 10 mg/L), and albumin (ALB < 35 g/L) [16]. Patients with cardiovascular diseases have been shown to have a significant increase in the risk of death when they are infected with SARS-CoV-2 (*p* < 0.001) [26].

Preliminary investigations on the impact of gender and sex on incidence, severity, and mortality of COVID-19 from China and Europe have indicated a gender imbalance with male predominance in disease prevalence, hospitalizations (>50%), and mortality [27,28]. 

### 2.1. Routes of Transmission

Epidemiological information has established that human-to-human transmission probably occurs primarily through respiratory droplets (coughing, sneezing, talking) and direct contact with an infected person [29]. Similarly and as with other respiratory infections, the possibility of COVID-19 and SARS-CoV-2 contamination through airborne/aerosol transmission in heavily exposed, crowded, and enclosed environments with insufficient ventilation is to be expected [30,31]. Besides respiratory transmission, other potential routes of SARS-CoV-2 transmission include the eyes [32], oral mucous membranes, and digestive tract (fecal–oral) [33]. A recent study reported that SARS-CoV-2 can survive in aerosols for three hours, and its stability on plastic and stainless steel is up to 72 h [29]. Guidelines from the Centers for Disease Control and Prevention (CDC) mention that the spread of COVID-19 through contact with contaminated inanimate surfaces and by touching one’s eyes, mouth, and/or nose is plausible but is now considered a less common way of transmission [6].

Currently, the CDC recommends physical distancing of at least 6 feet (2 m) between people indoors and outdoors to slow the rate of transmission of SARS-CoV-2. However, the available data suggest that the airborne transmission of infected droplets at typical indoor velocities can be greater than 6 feet (2 m), thus leading to the risk of infection existing beyond the recommended physical distancing [34,35].

### 2.2. Virus and Dentistry

In dental offices, routine procedures involving handpieces, ultrasonic/sonic instruments, and polishing/airway syringes in the presence of saliva and microbial deposits (plaque and calculus) generate splatter and bioaerosols. The particle sizes of the aerosols determine their dispersion and the viability of microbes within the droplets. Consistent with the 5 µm particle size cut-off based on CDC guidelines, aerosols are considered to contain droplets (>5 µm) and small particle droplet nuclei (<5 µm) [34]. Large droplets (>20–30 µm) are heavy and settle on the floor or surface after taking a ballistic trajectory [36]. In contrast, the small particle droplet nuclei (0.5–10 µm) can stay suspended in air for hours and can penetrate and lodge into the respiratory passages, thus transmitting this infection [35]. Past evidence has shown that aerosols are implicated in the transmission of SARS-CoV and influenza virus [8]. Dry surface and instrument contamination from coronaviruses leading to self-inoculation through mucous membranes is also plausible [37,38] and, therefore, poses an inherent risk of disease transmission to dental healthcare personnel.

The Occupational Safety and Health Administration (OSHA) report entitled “Guidance on preparing workplaces for Covid-19” classified dentistry as a very high exposure risk job, owing to involvement in aerosol-generating procedures [39]. With this definition in mind, it is critical for all dental offices to take mandated precautions to prevent risk of SARS-CoV-2 transmission while providing safe dental services to patients.

### 2.3. Staying Informed and Prioritizing

Guidelines from the CDC underscore both the need to prioritize dental services and the importance to provide adequate personal protective equipment (PPE) and supplies to support the procedures, considering the risk to patients from delayed care. Hospital readiness checklist tools recommended by WHO aid in assessing a hospital’s preparedness, capacity, and gaps regarding their response during the COVID-19 pandemic (Table 1) [40].

At the onset of the pandemic, the response by many dental practices was to open only for urgent and emergency visits, since dentists would be at high-risk. When providing emergency and urgent dental procedures, the CDC states that it is “crucial to minimize aerosol generation and the ensuing probability of transmission to personnel and patients” [41]. According to the American Dental Association (ADA), dental emergencies are defined as “potentially life threatening and require immediate treatment to stop ongoing bleeding or to alleviate severe pain or infection.” Dental emergencies include uncontrolled bleeding, cellulitis, and facial trauma that compromise the airway. Dental urgencies are considered severe pain from pulpal inflammation, third molar pericoronitis, fascial space infections of dental origin, trauma along with tooth avulsion, dental treatments prior to key medical procedures, management of alveolar osteitis, biopsies of abnormal tissues, and final crown or bridge cementation, if a temporary crown is displaced [42].

A study conducted on a cluster of patients who underwent surgery (under general, regional, or local anesthesia) and who had SARS-CoV-2 infection within seven days before or 30 days after surgery reported both post-operative pulmonary complications in half of the patients with peri-operative SARS-CoV-2 infection, and increased mortality. The study encouraged postponement of non-critical elective procedures and promoted non-surgical treatment options to delay the demand for surgery [43].

Currently, as dental facilities slowly begin to deliver elective dental services once again, it is crucial for the dental professionals to constantly analyze the data from their state and local healthcare departments to keep updated on zone and community-specific COVID-19 human transmission statistics and precautionary directives. 

### 2.4. Screening

In the evolution of the current pandemic, screening patients for COVID-19-related signs and symptoms prior to scheduling an appointment has emerged as an important tool to identify patients in need for isolation and also for contact tracing. Screening strategies include diagnostic testing, screening questionnaires, and temperature/symptom check protocols. The CDC has released a summary of consolidated recommendations for testing people for SARS-CoV-2 infection in certain situations:

1. Demonstrate signs and symptoms of COVID-19.
2. Have been in contact with people with suspected or confirmed cases of COVID-19 but are themselves asymptomatic.
3. Present no symptoms or had no known contact with suspected or confirmed COVID-19 individuals but still were tested by early identification in special settings.
4. Have been confirmed to have COVID-19 but are no longer symptomatic.
5. Were tested by public health officials for contact tracing or tracking the speed of COVID-19.

Source: CDC COVID-19 testing recommendations [6].

To date, the CDC has approved both viral and antibody tests for COVID-19. Viral tests determine the current SARS-CoV-2 infection status by checking nasal swabs. Antibody tests determine the past infection of SARS-CoV-2 by checking blood for antibodies against SARS-CoV-2. At present, a confirmatory diagnosis for COVID-19 is given by testing respiratory samples with a real-time polymerase chain reaction (RT-PCR) diagnostic panel [44]. Other testing advancements include using saliva samples for diagnostic detection of SARS-CoV-2 [45] and reverse-transcribed loop mediated isothermal amplification (RT-LAMP) protocols [46]. 

In dental practice, telehealth platforms can help assess the risk profile of a patient, needs, and aid in establishing a dental triage (Table 2).

Screening of patients before visiting the dental clinic will prevent exposure and spread of virus to clinical staff. At the same time, all the clinical staff should be screened every day for COVID-19 symptoms.

## 3. Prevention Control Strategies within Dental Offices 

Considering that SARS-CoV-2 has been consistently detected in saliva [48], this finding poses a challenge to dental health care professionals (DHCP) and emphasizes the need to prevent potential exposure to COVID-19 and to disrupt its transmission within dental facilities. When looking at present and future dentistry, it is important to understand that dental settings have unique aspects that require practices to have very specific and customized infection control methods. 

It is also imperative that employers and facility managers develop patient management strategies and overall practice controls (administrative, engineering, work practice controls) as shown in Table 3, that follow the highest standard-of-care. Employers need to ensure that dental facilities are totally well-prepared prior to resuming business and that all cross-functional staff are trained with access to all resources vital to preventing failures while also addressing COVID-19 challenges. 

Similarly, for optimal safety and protection, patient management strategies must be enforced before the dental appointment, upon patient arrival at the dental facility, during the procedure, and when leaving the dental facility. Recent data suggest that without social distancing or any other preventive measures, the risk of transmitting the coronavirus is 17.4%. However, a mask or respirator will reduce the risk of transmission to 3.1% [49]. 

### 3.1. Compiled COVID-19 Infection Prevention Guidance and Protective Measures for Dental Settings

1. Schedule appointments thoughtfully, ensuring limited number of patients in the clinic at any given time, and observing at least six feet of physical distancing.
2. Minimize the number of patients in the waiting area and personnel in the operating area at any given time.
3. Encourage patients to present alone, if possible, thus limiting the number of visitors in the clinic areas.
4. Advise patients and accompanying persons to arrive with a face covering or provide a surgical mask upon arrival.
5. Assess temperatures using a non-contact thermometer of all patients, visitors, and staff to rule out fever upon arrival.
6. Re-verify the screening questions verbally and through observation to rule out any COVID-19 signs and symptoms.
7. Ensure the waiting area is free of magazines, reading materials, and toys in order to limit the transmission through surfaces.
8. Install physical barriers (plastic or fiberglass) at the front desks to limit transmission in the reception area.
9. Post signs, posters, and flyers regarding COVID-19 recommendations and information addressing hand hygiene and cough etiquette in the dental care facilities for patient and staff education.
10. Educate patients on the possibility of prolonged appointments due to newer disinfection measures.
11. Implement contactless payments or pre-payments, thus reducing the number of interactions between patients and personnel.
12. Request patients to inform the clinic if they develop symptoms within 14 days of appointment.
13. Interim guidance developed by Public health agency of Canada recommends dental personnel to routinely practice point-of-care risk assessment (PCRA) prior to clinical interaction with the patient to assess the risk of infection exposure, high risk tasks and appropriate measures to minimize the potential exposure [19].
14. All dental personnel should adhere to standard infection control precautions, including personal protective equipment (PPE).
15. The CDC recommends health care workers to use N95 filtering facepiece respirators (FFR) that can filter at least 95% of all particles, including bacteria and viruses to a size of almost 0.3 µm. European standards classify filtering facepiece respirators (FFR) as FFP1 with minimum filtration efficiency (MFE) of 80%, FFP2 with MFE 94% and, FFP 3 with MFE 99%. European standard FFP2 are equivalent to N95 FFR’s [50]. Healthcare settings in United Kingdom recommend using European standard FFP 3 as the highest level of protection while performing high risk aerosol generating procedures [51].
16. Alternatives for N95 FFR include diverse options:
  (a) National Institute for Occupational Safety and Health (NIOSH) approved Filtering facepiece respirators: N99, N100, P95, P99, P100, R95, R99, and R100 [39].
  (b) An air purifying elastomeric respirator with appropriate filters and cartridges.
  (c) Powered air purifying respirators (PAPR): These are reusable battery powered respirators attached to a high efficiency particulate arrestor (HEPA) filter. PAPR provides better respiratory protection than N95 FFR. However, an ergonomic impact due to the weight of the filter can occur [52].
  (d) In the event of unavailability of a respirator for an aerosol generating procedure, the CDC recommends use of a surgical mask and a full-face shield [41].

1. Conduct a seal check of the FFR to determine whether the mask is properly worn, and types of seal checks include positive and negative pressure checks.
2. Dental healthcare personnel (DHCP) should limit clinical care to one patient at a time if possible.
3. Operating areas should be set up with only clean or sterile instruments. Any equipment that is exposed but not used during a procedure should be considered contaminated and should be disposed of or autoclaved properly.
4. Minimize aerosol-inducing and -generating procedures when possible and prioritize atraumatic restorations and minimally invasive procedures utilizing hand instruments only.
5. Minimize the number of intra-oral radiographs that could induce aerosols through coughing or gagging. Extra- over intra-oral imaging is preferred.
6. Aerosol generating procedures should be conducted in airborne infection isolation rooms (AIIR).
7. Airborne precautions should be employed when aerosol generating procedures are conducted. Four-handed dentistry is recommended using high vacuum suctions (HVE), rubber dams, and other appropriate appliances, such as extra-oral vacuum aspirators (EOVA) that can reduce infectious aerosol dispersion.
8. CDC guidelines mention the possibility of clinical effectiveness of preprocedural mouth rinses (PPMR) containing antimicrobial products, such as povidone-iodine, chlorhexidine, essential oils, and/or cetylpyridinium chloride for reducing the viral load in the aerosols and spatter. However, given that there are no adequate data published on the clinical effectiveness of preprocedural rinses on reducing viral titers of SARS-CoV-2, well-designed human trials are necessary to investigate this area of concern.
9. Engineering controls, such as maintaining ventilation systems in a clean to less clean air flow direction, HEPA air filtration units for enhanced air cleaning, and heating, ventilation, and air-conditioning (HVAC) systems for increased filtration efficiency should be considered.
10. The CDC recommends upper-room ultraviolet germicidal irradiation (UVGI) of wavelengths 200 and 280 nm as an adjunct to establishing adequate ventilation and air venting.
11. Implement strict hand hygiene measures before and after contact with patients, before donning and after removal of all PPE, and after contact with any potentially contaminated surfaces. Proper hand hygiene measures require using water and soap and alcohol-based hand rubs (60–95% alcohol) for at least 20 sec.
12. To clean and disinfect the operating area after a dental procedure, the CDC recommends a wait time of 15 min for air change to occur. Routine cleaning and standard disinfection should be conducted with an Environmental Protection Agency (EPA)-registered disinfectant. In a situation in which an aerosol generating procedure is conducted on a suspected or COVID-19 confirmed patient, the Australian Dental Association recommends “terminal/infectious clean” of the dental setting in which a two-step cleaning with detergent followed by hospital grade disinfectant is to be used [53].

(SOURCE: CDC Guidance for dental settings [34], Occupation Safety and Health Administration (OSHA) Guidance on preparing workplaces for COVID-19 [39], Interim guidance developed by Public health agency of Canada [19], Australian Dental Association guidance [53])

Administrative controls (or work practice controls) are changes in work procedures, such as written safety policies, rules, supervision, schedules, and training with the goal of reducing the duration, frequency, and severity of exposure to hazardous chemicals or situations. Engineering controls eliminate or reduce exposure to a chemical or physical hazard through the use or substitution of engineered machinery or equipment. PPE: Personal protective equipment. (Source: The MSDS Hyper Glossary). SOURCE: CDC: Strategies for optimizing the supply of N95 Respirators [52]).

(SOURCE: CDC Guidance for dental settings [34], Occupation Safety and Health Administration (OSHA) Guidance on preparing workplaces for COVID-19 [39]).

### 3.2. Managing the Availability of PPE/Supplies

Healthcare facilities have been advised to stock adequate PPE and related supplies to support patient volume, especially during expected and known shortages of essential supplies. Given the widespread scarcity of many medical necessities during the pandemic, the CDC recommends Contingency Capacity Strategies and Crisis Capacity Strategies [52]. Specifically, in cases in which dental care is concerned during a stage of contingency and crisis, dentists should decide how to provide treatment for the most vulnerable patients who present with emergencies and urgent issues.

### 3.3. Tiered Approach of Personal Protective Equipment (PPE)

With regards to PPE, Lockhart et al. proposed a three-tiered approach [54].

1. PPE for droplet and contact precautions.
2. PPE for airborne, droplet, and contact precautions.
3. PPE for airborne, droplet, and contact precautions for high-risk aerosol-generating medical procedures (AGMPs).

PPE for droplet and contact precautions is recommended for use during procedures that do not involve high-risk aerosol-generating medical procedures. This procedure includes several pieces of PPE: (1) surgical mask, (2) eye protection (goggles or procedure mask with face shield), (3) Association for the Advancement of Medical Instrumentation (AAMI) level-two gowns, and (4) gloves that overlap the gown sleeve.

PPE for airborne, droplet, and contact precautions is recommended for a healthcare provider present in the operating area during high-risk aerosol-generating medical procedures. This procedure includes several pieces of PPE: (1) N95 respirator, (2) eye protection (goggles or procedure mask with face shield), (3) head covering, (4) AAMI level-two gowns, and (5) gloves that overlap the gown sleeve.

PPE for airborne, droplet, and contact precautions for high risk AGMPs is recommended for healthcare providers directly involved in high-risk aerosol-generating medical procedures. This procedure includes several pieces of PPE: (1) N95 respirator, (2) eye protection (goggles or procedure mask with face shield), (3) head covering, (4) AAMI level two/three gowns, and (5) double gloves that overlap the gown sleeve. 

Lockhart et al. [54] recommends neck coverings while performing high-risk AGMPs as long as the covering does not restrict the operators’ movements. In fact, the neck area is a potential zone of contamination considering its proximity to mucous membranes that are located above this area. Donning and doffing PPE is critical, and all healthcare personnel and staff should be trained and be competent in the process. A study by Okamoto et al. reported that healthcare professionals made multiple errors while donning and doffing PPE [55]. A few errors included touching the inside of the gown or glove with a gloved hand, touching the outside of the gown or glove with bare hands, and not tightening the gown at the neck [55]. Studies suggest that doffing the PPE process poses a high risk of self-contamination [55,56] with a higher risk of hand contamination when gloves were removed before gown removal during PPE doffing [55]. Another study by Verbeek et al. suggests that having a “doffing spotter” read out the steps during the doffing PPE process reduces this risk [56]. A multinational study reported that the frontline healthcare workers (HCW) have a 12-fold increased risk of reporting a positive COVID-19 test relative to general community and healthcare workers with inadequate PPE exhibited a 31% increase in risk of contracting the virus compared to healthcare workers with adequate PPE [57]. This finding establishes the risk faced by healthcare professionals and stresses the need to practice and implement strict infection prevention and control protocols when caring for patients.

## 4. Guidance for Managing Treatment Procedures Involving High Risk Aerosol Generating Procedures (AGP)

Infectious aerosols generated from aerosol-generating dental procedures endanger dental personnel due to the risk of infection through inhalation, direct contact, and/or autoinoculation. The latest research characterizes the size of dental aerosols to be <0.3 µm (0.05–0.15 µm diameter range corresponding to the reported size of SARS-CoV-2 virus [0.05–0.15 µm]) and are estimated to be airborne for 28 to 34 min after the cease of dental aerosol generating procedures [58]. The research also states that high-volume intraoral suction alone or in combination with and air cleaning systems (ACS) containing HEPA filters are effective in reducing the airborne particle concentrations to baseline levels [58].

Ideally, the CDC recommends aerosol-generating dental procedures to be conducted on a single patient in a negative pressure AIIR [34]. The air from these rooms should either be filtered via HEPA filters before circulating or exhausted directly outside. Marlene et al. has provided an operative team checklist citing general principles of approach while performing high-risk aerosol generating procedures [59].

1. Take time to plan and to communicate modifications to usual approach.
2. Use highest level of PPE that is available (example: NIOSH approved, fit tested N95 FFR).
3. Limit the number of personnel to those who are necessary to safely perform the procedure.
4. Restrict operating area access once patient has entered the room.
5. Limit direct contact with patient as much as possible.
6. Employ the least number of aerosol-generating techniques and reduce open airway time.
7. Minimize handling of contaminated instruments and surfaces on which respiratory secretions may have settled.
8. Anti-retraction valves which prevent cross-contamination of the water.
9. Use of 3%hydrogen peroxide as mouthwash before procedure.

(Source: Marlene et al. [59])

## 5. Preprocedural Mouth Rinses against SARS-CoV-2

PPMR/gargle with PVP-I is considered effective in reducing the viral load of SARS-CoV [60] and MERS [61] in oral cavities and the oropharynx and in turn, alleviates viral load aerosolization. A review by Parhar et al. states that PVP-I in concentrations of 0.23% to 7% were viricidal against a diverse range of viruses, including SARS-CoV and MERS [62]. A recent study conducted by Bidra et al. suggests that PVP-I in concentrations of 0.5%, 1%, and 1.5% completely inactivated SARS-CoV-2 within 15 sec of contact. The study also found that 70% ethanol group could inactivate the virus after 30 s of contact with the ethanol [63].

## 6. COVID-19 and Oral Hygiene

Recent literature suggests the correlation between COVID-19 complications, oral health, and periodontal disease, and explores the possibility of reducing COVID-19-associated systemic complications by improving oral hygiene [64,65]. The comorbidities associated with an increase in the risk of complications and deaths from COVID-19 are linked with altered oral biofilms and periodontal disease, which can give rise to systemic inflammation, bacteremia, pneumonia, and even death [64]. Although the most important question that is still under speculation asks why some patients suffer from COVID-19 more severely than others, current data indicate that post-viral infection-related complications, such as acute respiratory distress syndrome (ARDS), are the main causes of death [64]. Scannapieco et al. proposed that oral bacteria play a potential role in the pathogenesis of respiratory infections via the action of multiple mechanisms [66]. Pathogenic bacteria and cytokines (such as interleukin-1 and tumor necrosis factor (IL-1 and TNF, respectively)) from diseased gums can infiltrate the saliva through the gingival crevicular fluid and be aspirated to cause lung infection [64]. Earlier studies have reported that in patients who are in the hospital or long-term care settings, pathogens isolated from lungs and oral cavity were genetically indistinguishable. These data suggest that inadequate oral hygiene can increase the risk of inter-bacterial exchanges between the lungs and the mouth, leading to a higher risk of having a respiratory infection. Contrastingly, improved oral hygiene can reduce the complications of pneumonia [65]. 

It is evident that the oral microbiome is an important determinant of lung microbiomes in both healthy and immunocompromised individuals. Importantly, a unique thought that has yet to be explored is the therapeutic alteration of the lung microbiome by changing the oral microbiome. Although much about the oral lung microbiome is yet to be understood, the authors have made it very clear that good oral health is beneficial and can help prevent the risks of being infected with systemic diseases. 

## 7. Future Forward

Considering the clinical significance of antibiotic misuse across healthcare fields, dentistry may indirectly impact COVID-19 by triggering deaths due to secondary infection [67]. According to the data, 50% of the COVID-19 patients who died had secondary bacterial infections [68]. Moreover, 66% of the selected antibiotics are not clinically indicated. Despite this fact, unnecessary antibiotic usage in dentistry still occurs and ultimately contributes to the growing problem of antibiotic resistance [69,70]. The danger of secondary infections and resistance to antibiotics are factors that make pandemics, such as COVID-19, so lethal [67]. This serious consequence of antibiotic misuse should serve as a caution for dentists to reconsider the prescription of antibiotics in their practices.

In the initial months of the COVID-19 pandemic, dental care along with other healthcare sectors came to a standstill. Now, in the wake of enhanced infection control practices and availability of adequate PPE, the dental care sector and dental economy have both been rebounding. In the United States, as of 15 February 2020, the ADA reports that patient volume to dental offices is recovering up to 81% of pre-COVID-19 levels, and dental staffing is stabilizing at 91% of pre-COVID-19 levels [71]. However, with the multiple variants of SARS-CoV-2, such as B.1.1.7, B.1.351, and P.1, which are originating and escalating globally, an urgent need for compliance and rigorous infection control strategies to curb the viral transmission in dental settings exists.

## 8. Conclusions

The COVID-19 pandemic is an unprecedented disaster that has significantly impacted the healthcare system, including dentistry. After analyzing the available data on SARS-CoV-2 and considering the fact of rapidly emerging trends in COVID-19 transmission, this study recognizes the multitude of implications regarding its spread, infection control, and prevention. This review article highlights relevant guidance and protocols recommended to mitigate SARS-CoV-2 transmission in dental facilities. In the wake of new data, it is expected that more innovative armamentarium and upgrades to infection control procedures in dental offices will be implemented [72,73]. In these extraordinary and unprecedented times, it is important for dental healthcare professionals to adhere to infection control protocols while providing services for patients in a safe environment.

## Figures and Tables

**Table 1:** World Health Organization (WHO) rapid hospital readiness checklist for Coronavirus 2019 (COVID-19). The checklist highlights 12 key components that are essential to managing COVID-19 in a hospital setting.

Leadership and incident management system
2. Coordination and communication
3. Surveillance and information management
4. Risk communication and community engagement
5. Administration, financial, and business continuity
6. Human resources
7. Surge capacity
8. Continuity of essential support services
9. Patient management
10. Occupational health, mental health and psychosocial support
11. Rapid identification and diagnosis
12. Infection prevention and control

Source: WHO Rapid hospital readiness checklist [40].

**Table 2:** Phone Advice and initial evaluation.

Telescreening Steps Include	Further Details
Evaluate symptoms	Symptoms include unexplained fever> 100.4 °F (or 38 °C), dry cough, shortness of breath, recent loss of taste/smell, sore throat
2. Exposure to a diagnosed COVID-19 individual	Duration: prior two weeks
3. Assess high-risk conditions	This assessment includes immunocompromised patients
4. Assess special circumstances	This includes patients living in nursing homes/long-term care facilities

Source: CDC Phone Advice Line Tool for possible COVID-19 patients [47].

**Table 3:** Consolidated review of administrative, engineering controls, and PPE guidelines recommended by the Occupational Health and Safety Administration and the Centers for Disease Control (OSHA and CDC, respectively).

Administrative Controls	Engineering Controls	PPE
Limiting the number of personnel in the hospital at any given time Regular monitoring of staff for COVID 19 symptoms Encouraging suspected/confirmed COVID 19 staff to quarantine Cohorting patients with confirmed COVID-19 infections. Cohorting healthcare professional (HCP): Assigning designated team of HCPs to provide care for suspected/confirmed COVID-19 patients. Training the staff on PPE competency ahead of patient consultation Training on the use of N95 FFR and Qualitative fit testing Adopting just-in-time fit testing. Providing up to date education to hospital staff concerning COVID 19 risk factors and protective measures Employing safe work practices such as access to alcohol-based sanitizers, PPE, EPA approved disinfectants and regular cleaning equipment. Posting signs and flyers about hand hygiene, and cough etiquette in the hospital.	High efficiency air filters Improving ventilation Negative pressure rooms/Airborne Infection Isolation Rooms (AIIR) for Aerosol Generating Procedures (AGP) Physical Barriers Ozone generating devices for air purification Antimicrobial Irrigants system Anti-retraction valves which prevent cross-contamination of the water	Disposable gowns Gloves Eye protection Face shields Facemasks: FFP 2/FFP3/PAPR Additional PPE: Hair covers Shoe covers
